# Impact of Platform Design and Usability on Adherence and Retention: Randomized Web- and Mobile-Based Longitudinal Study

**DOI:** 10.2196/50225

**Published:** 2025-03-27

**Authors:** Xinrui Jiang, Michelle Timmons, Elias Boroda, Marie Onakomaiya

**Affiliations:** 1 Google via Akraya, Troy, MI, United States; 2 Hackensack Meridian Health, Hackensack Meridian School of Medicine, Nutley, NJ, United States; 3 Datacubed Health, 630 Freedom Business Center Dr, 3rd Floor, King of Prussia, PA, 19406, United States, 1 (302) 6350830; 4 Metric Health, New York, NY, United States

**Keywords:** behavioral science, electronic patient-reported outcomes, ePROs, retention, adherence, patient engagement, clinical trials, mobile phone

## Abstract

**Background:**

Low retention and adherence increase clinical trial costs and timelines. Burdens associated with participating in a clinical trial contribute to early study termination. Electronic patient-reported outcome (ePRO) tools reduce participant burden by allowing remote participation, and facilitate communication between researchers and participants. The Datacubed Health (DCH) mobile app is unique among ePRO platforms in its application of behavioral science principles (reward, motivation, identity, etc) in clinical trials to promote engagement, adherence, and retention.

**Objective:**

We evaluated the impact of platform design and usability on adherence and retention with a longitudinal study involving repeated patient-facing study instruments. We expected participants assigned to complete instruments in the DCH mobile app to stay in this study longer (increased retention) and complete more surveys while in this study (increased adherence) due to the enhanced motivational elements unique to the participant experience in the DCH app group, and this group’s overall lower burden of participation.

**Methods:**

A total of 284 adult participants completed 24 weekly surveys via 1 of 4 modalities (DCH app vs DCH website vs third-party website vs paper) in a web-based and mobile longitudinal study. Participants were recruited from open access websites (eg, Craigslist or Facebook [Meta]), and a closed web-based user group. All participation occurred remotely. Study staff deliberately limited communications with participants to directly assess the main effects of survey administration modality; enrollment and study administration were largely automated. Participants assigned to the DCH app group experienced behavioral science–driven motivational elements related to reward and identity formation throughout their study journey. There was no homolog to this feature in any other tested platform. Participants assigned to the DCH app group accessed study measures using passcodes or smartphone biometrics (face or touch ID). Participants in the DCH website group logged into a website using a username and password. Participants in the third-party website group accessed web-based surveys via personalized emailed links with no need for password authentication. Paper arm participants received paper surveys in the mail.

**Results:**

Mode of survey administration (DCH app vs DCH website vs third-party website vs paper) predicted study retention (*F*_9,255_=4.22, *P*<.001) and adherence (*F*_9,162_=5.5, *P*<.001). The DCH app group had greater retention than the paper arm (*t*=−3.80, *P*<.001), and comparable retention to the DCH website group. The DCH app group had greater adherence than all other arms (DCH web: *t*=−2.42, *P*=.02; third-party web: *t*=−3.56, *P*<.001; and paper arm: *t*=−4.53, *P*<.001).

**Conclusions:**

Using an ePRO platform in a longitudinal study increased retention and adherence in comparison to paper instruments. Incorporating behavioral science design in an ePRO platform resulted in further increase in adherence in a longitudinal study.

## Introduction

Clinical trial retention and adherence rates vary greatly across and within therapeutic areas [1-3]. Low adherence and retention increase costs and negatively impact data quality and the validity of research findings. Mitigating the various retention and adherence challenges in clinical trials is a major focus of clinical trial sponsors and researchers [4]. Studies can improve retention by strategically recruiting individuals or populations more likely to complete a trial [1]. However, this can increase the risk of bias and decrease the degree of representativeness in the study sample. Retention challenges can especially impact at-risk populations, an effect that increases with study duration [5]. Thus, preselecting participants based on their likelihood of completing a longitudinal clinical trial may not best represent the targeted indication itself. Further, patterns of risky behavior may predict dropout as seen in bipolar disorder and adolescent depression treatment studies [6,7].

Researcher behavior and communication also impact participant retention. Retention increases with participants’ positive attitudes toward study staff and the quality of their relationship with the study team [8,9]. Focusing on patient-centered communication and relationship building can therefore bolster retention in a clinical trial, but is not necessarily effective for all study designs and populations [4]. Participant burden further impairs study retention; the more difficult or inconvenient it is to participate in a study, the more likely participants are to stop participating [10]. The sources of participant burden vary with study design and indication. Common examples include longer trial duration, protocol complexity, financial difficulties, and travel-related burden [10-12].

eCOAs (electronic clinical outcome assessments) such as electronic patient reported outcomes (ePROs) and electronic diaries are popular ways to incorporate the patient perspective and reduce participant burden in clinical trials [13]. ePRO platforms vary in their design attributes and usability, and different study populations have different aesthetic and performance preferences [14,15]. Regardless, participants across diverse indications report high usability and tolerability of ePRO platforms [16-18]. In comparison to paper data collection, ePRO platforms improve timeliness of questionnaire delivery, minimize data entry errors, and reduce cognitive burden for study participation by automating reminders. Some ePRO platforms allow researchers to communicate with participants, fostering the development of a personal connection with the study team that has been associated with increased study retention [8,9]. Through creating an easier experience for participants, these features increase adherence and retention, a goal shared by all clinical research studies. Further, participants otherwise lost to follow up may continue providing data, if they have the option to do so remotely [18].

However, ePRO platforms have unique challenges that impact retention and adherence. Older adults are particularly concerned about security and data sharing with electronic platforms [19]. Regulatory guidelines often mandate that researchers prioritize data security when selecting an ePRO platform. Maximizing data security can increase participant burden by requiring complex passwords or additional security measures such as 2-factor authentication [20]. Researchers consequently have multifaceted challenges to contend with when designing a study that ensures ease of participation, while simultaneously complying with good clinical practice standards and maximizing data security.

**Figure 1.**
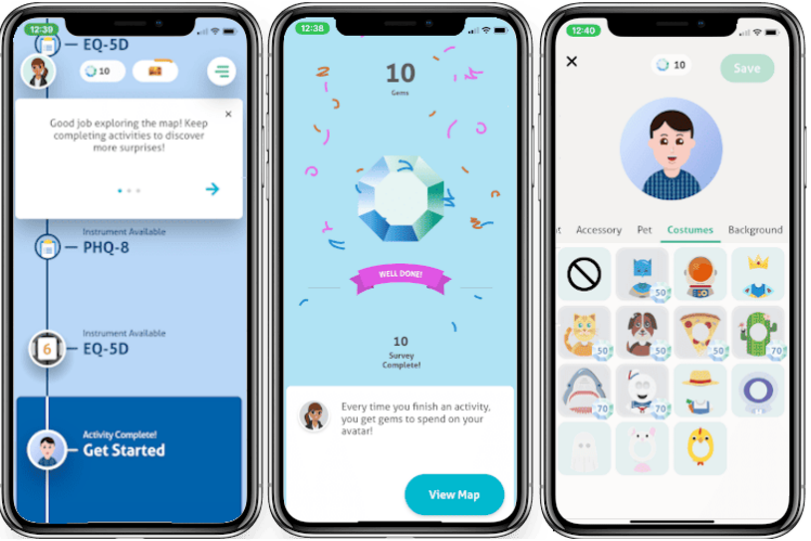
Behavioral science-based design of the DCH app. Participants assigned to complete surveys using the DCH app encountered in-app motivators and rewards throughout their study journey. DCH: Datacubed Health.

Datacubed Health (DCH) offers one such ePRO platform. It is differentiated from other platforms by its behavioral science-focused user experience design and in-app motivational elements (Figure 1). In general, mobile app users report higher consumer loyalty and more positive attitudes toward core services when app usage involves reward, achievement, gaining knowledge, and identity formation [21]. ePRO platforms, which leverage these principles in their design, may especially maximize retention and adherence in clinical trials [22,23]. Participants using the DCH app achieve a sense of identity by creating an in-app avatar to represent them. As participants progress through the study, they are rewarded for completing study activities. Participants’ progress is visualized dynamically, contributing to a sense of achievement. At the study level, researchers may choose to deploy educational materials about this study, treatment, or indication, allowing participants to gain knowledge. Together, these features encourage continued retention and adherence by fostering a positive attitude toward study participation. Previous studies using DCH’s ePRO system have achieved high adherence (eg, 100% in [24]) and retention (eg, 93.5% in “virtual trials” [25]).

This study evaluated the impact of behavioral science–based ePRO platform features on adherence and retention in a longitudinal virtual study involving weekly completion of questionnaires for 6 months. Further, 3 ePRO platforms (DCH app, DCH website, and a third-party website) were compared to each other and to the traditional paper survey administration. We hypothesized that reducing friction and increasing motivation by administering ePROs using DCH’s behavioral science-based mobile app would result in higher adherence and retention beyond the benefits of ePROs without these functions (ie, DCH website and third-party website).

## Methods

### Ethical Considerations

This study was conducted under institutional review board (IRB) approval from the BRANY (Biomedical Research Alliance of New York; #20-017-740) and the protocol is publicly available (DOI: 10.5281/zenodo.14807237) or available as Multimedia Appendix 1. All participants reviewed and completed informed consent in the DCH app using the Health Insurance Portability and Accountability Act and General Data Protection Regulation compliant eConsent feature of the app. Participants were required to answer challenge questions during the consent process to ensure they understood participation requirements. While participants provided their contact information to participate in this study, the dataset and all reported findings were deidentified before analysis. Participants were compensated US $5 for each survey they completed during this study. Payment schedule varied as an outcome measure as described further below.

### Participant Recruitment

Participants were recruited from advertisements placed on open access websites including Craigslist, Facebook, and Snapchat. A subset of participants was recruited using the services of a closed user group, a participant recruiting platform for user experience research. Recruitment was fully automated; advertisements contained a link to the screening survey. Participants who met screening criteria received an automated email invitation to download the DCH app and a unique code to create an account within the app for informed consent. All participants reviewed the informed consent form remotely, via DCH’s electronic consent module. Consent comprehension questions were required before electronic signature to ensure participants understood this study’s requirements and duration. In order to complete eConsent procedures, participants were required to download the DCH app onto their personal smartphone device, and required to share a minimum of necessary data with the DCH app developers. There was not a possibility of individual data being bequeathed to or sold to third parties, with or without participant consent.

### Eligibility Criteria

Participant demographics were unknown to researchers during recruitment in the interest of recruiting a diverse, heterogeneous set of participants. However, to facilitate study participation and comply with IRB requirements, we excluded participants who self-reported that they did not have access to a smartphone, did not have a data plan, did not reside within the United States, were younger than 18 years, or did not speak English fluently. We further excluded participants whose IP address indicated they did not reside within the United States, or who were using IP spoofing software. We excluded participants who used the same IP address to complete the automated, web-based screening process multiple times; these participants were able to enroll in this study only once, provided they otherwise met eligibility criteria.

### Participant Demographics

Participants completed a self-reported demographics questionnaire in their assigned administration modality during their first week of participation. Participants were on average aged 34.78 (SD 12.79) years and mostly identified as female (n=149, 54.18%) or male (n=116, 42.18%) from diverse racial or ethnic backgrounds (Table 1). A total of 180 participants were retained for the full 6-month study duration, meaning they completed the final or week 24 survey. Adherence was assessed based on data from these retained participants.

**Table 1.** Participant demographics. A total of 284 participants were randomly assigned to complete weekly surveys using 1 of 4 modalities (DCH^a^ app vs DCH web vs third-party website vs paper). A total of 275 of these participants completed a survey providing their demographic data.

Demographics		Values
Age (year), mean (SD)		34.78 (12.79)
**Gender identity, n (%)**		
	Female	149 (54.18)
	Male	116 (42.18)
	Gender queer or gender nonconforming	8 (2.91)
	Prefer not to say	2 (0.73)
**Race or ethnicity, n (%)**		
	Asian	46 (16.73)
	Black or African American	37 (13.45)
	Hispanic or Latino	18 (6.55)
	White	149 (54.18)
	More than 1 race	20 (7.27)
	Other race	4 (1.45)
	Prefer not to say	1 (0.36)

a DCH: Datacubed Health.

### Randomization

A total of 284 participants were randomly assigned to receive weekly surveys via 1 of 4 modes of administration (DCH app vs DCH website vs third-party website vs paper). Participants were assigned sequentially, based on the order in which they completed the automated screening and consent procedures. Due to the nature of this study, participants were not blinded and were aware of which mode of administration they were assigned to for the duration of this study. Similarly, study staff were not blinded. However, study staff interactions with participants were limited to IRB required communication, and mostly involved payment coordination via email.

### Survey Administration

After randomization, participants received email instructions corresponding to their study arm assignment (Table 2). All surveys were completed remotely by participants without monitoring or intervention by study staff. Surveys were selected to be easy to complete with neutral subject matter, such as the Perceived Stress Scale [26] and Patient Health Questionnaire-8 [27]. While the majority of surveys used were standard, validated ePROs, we developed a novel survey (“Format Usability Survey”) for this study to assess tolerability between different modes of administration, deployed at 3 time points throughout this study to all participants (weeks 4, 11, and 23). The Format Usability Survey included 30 items related to participants’ assigned platform (eg, “The format is easy to use” or “The format is user friendly”) rated on a 7-point Likert scale ranging from “strongly disagree” to “strongly agree,” and 2 open-ended prompts in which participants listed the positive or negative aspects of their assigned platform.

**Table 2.** Modes of survey administration and authentication. A total of 284 participants were randomly assigned to complete weekly surveys via 1 of 4 modalities (DCH^a^ app vs DCH website vs third-party website vs paper). These platforms differed in their modes of survey deployment and authentication.

Arm	Survey deployment	Authentication
DCH app	DCH app, with optional automated push notifications^b^	Username and password, smartphone biometrics, or passcode^c^
DCH website	Single email containing link to DCH website	Username and password
Third-party website	Single email containing link to third-party website	None
Paper	Mailed packets containing survey and stamped return envelope	None

a DCH: Datacubed Health.

b Participants were given the option to opt out of Datacubed Health app push notifications, if preferred.

c The Datacubed Health app can be configured to prompt participants to enable biometric authentication (eg, touch or face ID) after they first log-in with a username and password. Participants then create a numeric passcode. Participants may opt out of enabling biometric authentication and use only a passcode, if preferred.

### Participant Communication

#### Survey Response Monitoring

Throughout the 6-month study duration, this study’s team never proactively contacted study participants to remind them to complete surveys or encourage adherence. In general, communication with study participants across all arms was deliberately limited to assess the adherence capabilities of the 4 platforms without any confounds related to this study team’s encouragement or involvement. Participants were provided with a study email address for any necessary communications (eg, questions about payment or requests for study withdrawal).

Survey response monitoring was not conducted in this study as the main goal was to evaluate the impact of survey administration format on retention, adherence, and engagement in a virtual community population. This was communicated with all participants in the informed consent form.

#### DCH App

Participants assigned to the DCH app arm received weekly surveys in the DCH app, which they had already downloaded to complete the consent process. Participants could log into the DCH app by using smartphone biometrics (face or touch ID) or a 4-digit passcode. Participants in the DCH app arm who enabled push notifications received automated push notifications reminding them to complete surveys on a weekly basis. Participants were given the option to opt out of push notifications at study start, or were free to turn them off in their smartphone settings at any point throughout this study. Additional motivational elements unique to the DCH app arm included various in-app rewards for completing surveys and making progress.

#### Participatory Involvement

The DCH app was developed using behavioral science research, focus groups, and surveys over several iterative rounds of user experience testing spanning several years [28]. At the time of study conduct the DCH app was in use commercially as a patient-facing ePRO platform for international clinical trials. Before deployment for an individual clinical trial or research study, the DCH app undergoes a study-level user acceptance testing (UAT) protocol in which sponsors evaluate both the patient and sponsor or site-level experiences within the DCH app. The UAT process can occasionally identify bugs in the patient-facing experience, which are then promptly fixed, sometimes involving the release of new versions in the Google Play or Apple App stores. Notably, backward compatibility is maintained such that older app versions remain functional. For this study, UAT was performed by study staff before enrolling the first study participant.

At study start, participants were able to download version 3.50.5 (Android; Google) or 3.50.4 (iOS; Apple) from the Google Play or Apple App store, respectively. Both Android and iOS versions of the DCH app were continuously updated throughout this study when absolutely necessary; for example, for major bug fixes needed to maintain functionality. However, the DCH app did not undergo major changes during study conduct and all relevant participant-facing motivational features (eg, avatars or rewards) remained constant for the duration of data collection. The DCH app is Health Insurance Portability and Accountability Act and General Data Protection Regulation compliant with appropriate security and privacy measures in place to encrypt and protect participant data during and after their participation.

#### Reporting Guidelines

This study was reported referencing the CHERRIES (Checklist for Reporting the Results of Internet E-Surveys) and CONSORT (Consolidated Reporting of Standardized Trials) guidelines [29,30].

#### DCH Website and Third-Party Website

Participants assigned to the DCH website or third-party website arm were instructed to delete the DCH app, and received weekly emails containing links to web-based surveys hosted on the DCH website or the third-party website, respectively. The third-party website arm clicked email links to complete questionnaires directly. The DCH website arm clicked email links, then entered a unique username and password to access the surveys each week.

#### Paper

Participants assigned to the paper arm were prompted to enter their mailing address in the DCH app they had used to give consent, and upon doing so were instructed to delete the app and informed they would receive mailed surveys going forward. There was no authentication associated with completing paper surveys. Participants in the paper arm received weekly paperboard mailers containing a stamped reply envelope with which to return their completed surveys.

### Participant Compensation

All participants received US $5 via electronic transfer for each completed survey (Table 3). However, payment schedule varied to account for potential effects on adherence and retention for the paper arm participants whose mailed surveys needed to be returned and processed before compensation. This was of particular concern as data collection principally occurred during the height of the COVID-19 pandemic’s impact on US Postal Service delays [31]. Therefore, approximately one half of participants (n=161, 56.7%) received biweekly payments of US $5 per survey completed within the previous 2 weeks (biweekly), and the other half (n=123, 43.3%) received 1 lump sum payment for all completed surveys at the end of their 6 months in this study or request to withdraw from this study early (bulk). All participants were eligible to receive a maximum of US $120 corresponding to 24 completed surveys, or 6 months of weekly surveys.

**Table 3.** Participant groups by study arm and payment group. A total of 284 participants were randomly assigned to complete weekly surveys via 1 of 4 modalities (DCH^a^ app vs DCH website vs third-party website vs paper). Participants were further split into receiving ongoing payment for their study participation (biweekly) or 1 large payment upon their completion of this study (bulk).

Arm	Biweekly payment (biweekly)	One payment at study completion (bulk)
DCH app (n=95)	55	40
DCH website (n=45)	30	15
Third party website (n=88)	49	39
Paper (n=56)	27	29

a DCH: Datacubed Health.

### Statistical Analysis

Descriptive statistics were evaluated for each of the 4 study arms. Multiple linear regressions with dummy coded categorical independent variables were performed to examine the effect of survey modality (DCH app vs DCH website vs third-party website vs paper), payment schedule (bulk vs biweekly), and demographic variables (ie, age, gender, and ethnicity), on the primary outcome measures of retention (number of days between the first and last completed surveys) and adherence (percentage of surveys completed). Retention was defined as remaining in this study for the entire, 6-month duration, regardless of the number of surveys completed in that time period. Adherence was defined as the proportion of surveys completed while enrolled in this study. The adherence analysis set was restricted to participants who were retained till the study end, that is, completed the last survey (n=172). All statistical analyses were conducted using RStudio (Posit PBC) [32].

## Results

### Recruitment

**Figure 2.**
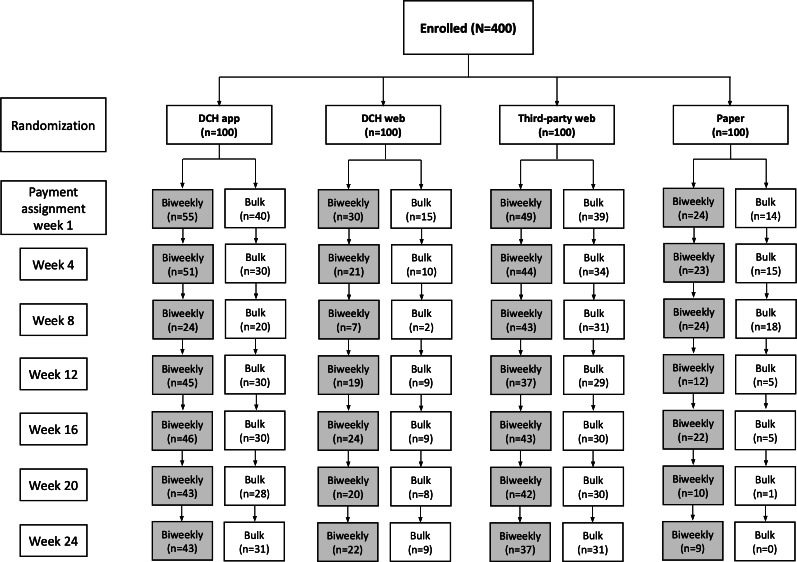
Enrollment and group assignment. A total of 116 participants left this study before completing a single survey. Further, 284 participants were included in the analysis dataset. DCH: Datacubed Health.

The analytic dataset included 265 participants, with 91 in the DCH app group, 45 in the DCH web group, 81 in the third-party web group, and 48 in the paper arm (Figure 2). For each group, 100 participants were recruited at baseline. Discrepancies in the number of participants in each group are attributable to differences between each study modalities’ tolerability to participants and subsequent attrition (eg, high attrition in the paper arm). This was expected and is directly relevant to this study’s primary outcomes of the impact of differences in retention and adherence based on the mode of survey administration. Participants were recruited between August 2020 through July 2021, and all individuals participated for a maximum of 6 months of follow-up.

### Baseline Data

Descriptive statistics for demographic variables across each study arm are reported in Table 4.

**Table 4.** Participant demographics by study arm. A total of 10 (3.64%) participants who reported their gender as “other” or “prefer not to say” were excluded for the purposes of analyses. Ethnicity groups of “more than 1 race,” “hispanic or latino,” “other race,” and “prefer not to say” were merged as 1 “other” group due to small sample sizes for the purposes of analyses.

		Study arm			
		DCH^a^ app	DCH web	Third-party web	Paper
Age (year), mean (SD)		34.99 (12.34)	35.38 (15.06)	34.59 (13.16)	35.73 (11.27)
**Gender, n (%)**					
	Female	50 (54.95)	26 (57.78)	46 (56.79)	27 (56.25)
	Male	41 (45.05)	19 (42.22)	35 (43.21)	21 (43.75)
**Race or ethnicity, n (%)**					
	Asian	13 (14.29)	10 (22.22)	14 (17.28)	8 (16.67)
	Black or African American	16 (17.58)	4 (8.89)	11 (13.58)	5 (10.42)
	Hispanic or Latino	8 (8.79)	6 (13.33)	3 (3.7)	—^b^
	White	48 (52.75)	22 (48.89)	47 (58.02)	29 (60.42)
	More than 1 race, prefer not to say, or other	6 (6.59)	3 (6.67)	6 (7.41)	6 (12.5)

a DCH: Datacubed Health.

b Not available.

### Multiple Regression Results

#### Overview

Predictors of retention (Table 5) and adherence (Table 6) were examined using multiple regression. Before analysis, assumptions were evaluated including linearity (residuals vs fitted), normality (Q-Q residuals), homoscedasticity (scale-location), and influential outliers (residuals vs leverage). All assumptions were met except normality. While violations of normality were identified in both cases, considering the large enough sample size we proceeded with analyses without modifying the dataset.

**Table 5.** Predictors of retention.^a^

Independent variable	β value	Standard error	*t* test^b^	*P* value
**Study arm** ^**c**^				
DCH^d^ web (vs DCH app–)	−18.34	10.5	–1.75	.08
Third-party web (vs DCH app)	4.26	8.75	0.49	.63
Paper (vs DCH app)	–38.99	10.27	–3.8	<.001
**Payment schedule**				
Biweekly (vs bulk)	25.05	7.26	3.45	.001
Age (years)	0.14	0.29	0.49	.625
**Gender**				
Male (vs female)	1.24	7.16	0.17	.86
**Ethnicity**				
Asian (vs White)	10.24	10.16	1.01	.31
Black or African American (vs White)	–10.8	10.69	–1.01	.31
Other (vs White)	4.11	10.53	0.39	.70

a *R*2=0.13, adjusted *R*2=0.10. *F*_9.255_=4.22, *P*<.001.

b 2-tailed.

c Reference groups are included in parentheses where applicable.

d DCH: Datacubed Health.

**Table 6.** Predictors of adherence.^a^

Independent variable		β value	Standard error	*t* test^b^	*P* value
**Study arm**					
	DCH web (vs DCH app)	–3.72	1.54	–2.42	.02
	Third-party web (vs DCH app)	–4.38	1.23	–3.56	<.001
	Paper (vs DCH app)	–12.4	2.74	–4.53	<.001
**Payment schedule**					
	Biweekly (vs bulk)	–1.38	1.14	–1.21	.23
Age (years)		–0.05	0.04	–1.2	.23
**Gender**					
	Male (vs female)	2.69	1.11	2.43	.02
**Ethnicity**					
	Asian (vs White)	–3.51	1.57	–2.24	.03
	Black or African American (vs White)	–0.09	1.66	–0.06	.955
	Other (vs White)	–1.2	1.6	–0.75	.453

a *R*2=0.23, adjusted *R*2=0.19; *F*_9,162_=5.5, *P*<.001.

b 2-tailed.

#### Retention

The overall retention model was statistically significant (*F*_9,255_=4.22, *P*<.001, *R^2^*=0.13, adjusted *R^2^*=0.10). The DCH app had greater retention than the paper arm (*t*=–3.80, *P*<.001). Biweekly payment schedule predicted greater retention than bulk payment (*t*=3.45, *P*=.001).

#### Adherence

The overall adherence model was statistically significant (*F*_9,162_=5.5, *P*<.001, *R^2^*=0.23, adjusted *R^2^*=0.19). The DCH app arm had superior adherence to the other 3 study arms (ie, DCH web, *t*=−2.42, *P*=.017; third-party web *t*=−3.56, *P*<.001; and paper arms, *t*=−4.53, *P*<.001). Male participants had significantly greater adherence than female participants (*t*=2.43, *P*=.02). Participants who identified as Asian had significantly lower adherence compared to participants who identified as White (*t*=−2.24, *P*=.03).

## Discussion

### Principal Findings

We examined the effect of ePRO platform design on longitudinal retention and adherence in a siteless, virtual study involving weekly questionnaires in a sample of 284 US-based adults. Compared to paper administration, ePROs, when paired with rewards, have been shown to improve retention and adherence in clinical settings [18,22,23]. This study specifically examined the impact of behavioral science elements in the DCH ePRO platform (eg, rewards for completing instruments, gamification, or automated reminders) on retention and adherence, compared to web-based ePRO platforms without motivators, and paper. We expected participants assigned to complete weekly instruments in the DCH app to show higher adherence and retention, due to the added motivational elements and lower friction intrinsic to the DCH app.

As expected, mode of administration significantly impacted both adherence and retention (*P*<.001). The DCH app had significantly higher retention than the paper format (*P*<.001) and significantly greater adherence than the other 3 study arms (ie, DCH web, *P*=.03; third-party web and paper arms, *P*<.001). While the retention rate for the third-party website was similar to that of the DCH app, participant-level authentication is a general standard for ePRO completion in clinical research, limiting this tools’ in vivo relevance for clinical trial use. Importantly, the DCH app arm, with secure authentication measures, had comparable retention to the third-party website, which had no authentication measures. These results suggest that unlike requiring a username and password, passcodes and biometric authentication are well tolerated security mechanisms that do not increase attrition in longitudinal studies.

The significant difference in adherence, but comparable retention, between the DCH app and third-party website arms suggests that differences between the 2 platforms contributed to higher overall adherence in the DCH app arm. The standard DCH app participant experience involves creating a representative avatar to build identity. As participants complete sequential surveys, they accumulate rewards and encounter various in-app motivators throughout this study’s journey. In addition, the user interface uses dynamic, colorful changes and progress markers. In comparison, the third-party website has no indicators of overall study progress or explicit motivators; participants simply click an email link to directly complete a survey. When used in clinical trials, apps like the DCH app allow study staff to enact more focused and immediate intervention in situations jeopardizing data completeness, for example, missing data, attrition, or app crashes in comparison to external website or survey platforms.

Among the examined demographic variables (ie, gender, ethnicity, or age), gender and ethnicity were significantly associated with adherence. Male participants showed significantly greater adherence (*P*=.02). However, the significance of this finding requires further exploration, ideally with a sample inclusive of nonbinary gender identities which were underrepresented in this study, and not reflected in the regression analysis. Participants who identified as Asian had lower adherence than participants who identified as White (*P*=.03). Future research can evaluate the meaning of these differences by recruiting a sample with expanded variability across gender and ethnicity groups.

To determine the impact of financial compensation on retention and adherence, participants were divided into 2 groups with different payment schedules. The results revealed that while the biweekly schedule was associated with greater overall retention than the bulk method (*P*=.001), payment schedule was not associated with adherence (*P*=.23) among those retained by study end. It is possible that restricting analyses to participants retained by study end represents a unique subgroup of individuals from the complete study sample.

Indeed, participants assigned to the paper arm were more likely to drop out if they also needed to wait 6 months to receive any compensation, such that 0 participants assigned to the paper arm with bulk payment schedule were retained to this study’s end. Delays and friction intrinsic to paper survey completion account for the low retention in the paper arm overall. In the absence of regular financial compensation, the burdens appeared to outweigh the delayed benefit for those in the paper arm. Qualitative data from paper arm participants in the Format Usability Survey support this assertion (eg, “May require trip to the post office to send out …… If using pen and a correction needs to be made. White-out may need to be used, which is kind of a hassle.” Additionally “Cumbersome especially if several pages, requires extra steps of sealing in envelope and dropping off in mailbox, writing is slower than typing.”). Future research evaluating the interaction between study participation burden and payment schedule is needed to confirm this hypothesis. While this study found no significant impact of regular versus bulk study payments for the electronic arms, this could change with increased participation burden. This is important when weighing the choice of administrative burden (eg, weekly payments) and participant retention.

While not assessed in this study, using paper to collect patient reported outcome measures adds significant additional site and sponsor-facing burden. Paper responses must be entered into an electronic record, a complex process which not only adds administrative burden and prolongs timelines but importantly introduces the opportunity for human error to alter study results (eg, data entry errors). In turn, the process of correcting data entry errors creates further administrative burden. Using electronic methods of data collection mitigates much of the delay and opportunity for data errors associated with paper data collection.

### Limitations

Participant notification within the DCH app arm varied based on individual preferences, because participants could opt out of push notifications alerting them to new or incomplete surveys. The DCH app arm was the only condition with the possibility for variability in notifications, but was also the only arm with any automated reminders. Other in-app motivators (eg, rewards or participant avatar) were equally available to all participants in the DCH app arm. Participants could not be blinded to their own arm assignment because survey administration platforms were this study’s arms.

Differences between the DCH app and third-party website were not strictly limited to additional behavioral science elements within the DCH app since 1 was a mobile app and the other a website. Ideally, 2 identical app-based platforms that differ only in their use of behavioral science elements (eg, rewards, avatars, etc) would be compared to confirm with greater confidence the incremental impact of behavioral science elements on study retention and adherence. In this case, other ways (eg, being a mobile app instead of a website or the intuitive design of the app interface) in which the DCH app improved upon the overall user experience of the third-party website may have contributed, at least in part, to the increased adherence seen in the DCH app arm. We were unable to comprehensively address several essential aspects of electronic health studies such as average session length due to this study’s design and lack of availability of an equivalent, comparable metric across the 4 platforms. Follow-up studies could incorporate these variables in their design.

Overall retention rates were somewhat low in this study, likely a consequence of this study’s design. Researcher communication impacts retention [4,9,10], so we deliberately limited communication with participants to isolate the main effect of survey platform on retention and adherence. In clinical trial settings, researchers commonly contact participants at risk of dropout proactively, which is an important complement to the use of technology. Regardless, the retention differences between study arms enforce the benefits of low-friction platforms.

### Conclusion

These results support the superiority of electronic administration over paper when conducting longitudinal data collection. However, not all ePRO platforms are equal; platform-level differences in participant-facing friction and motivators are associated with differences in retention and adherence, respectively. Specifically, reducing participant friction when logging in to an ePRO platform can promote retention. Longitudinally, participants were most willing to continue using platforms with lower-friction authentication methods, such as face or touch ID, in comparison to needing to remember and repeatedly enter a username and password. Additionally, the platform with behavioral science-based motivational features had significantly higher adherence than any other modality in this study, suggesting efficacy for long-term studies. Low retention and adherence pose a significant challenge to clinical research conduct, increasing the time and costs required to bring novel interventions to patients who need them. By choosing ePRO platforms that make participation in clinical trials easier and more enjoyable for participants, researchers can reduce costs, minimize site burden, and maximize participant benefit by accelerating clinical trials. Clinical trial sponsors and study teams should consider the patient experience when selecting an ePRO platform.

## Supplementary material

10.2196/50225 Multimedia Appendix 1 Study protocol.

## Abbreviations

BRANY: Biomedical Research Alliance of New York
CHERRIES: Checklist for Reporting the Results of Internet E-Surveys
CONSORT: Consolidated Reporting of Standardized Trials
DCH: Datacubed Health
eCOA: electronic clinical outcome assessment
ePRO: electronic patient reported outcome
IRB: Institutional Review Board
UAT: user acceptance testing
