# Supplementary material for: Impact of Platform Design and Usability on Adherence and Retention: Randomized Web- and Mobile-Based Longitudinal Study
Source: J Particip Med. 2025 Mar 27;17:e50225. doi: 10.2196/50225 (PMC11967695; doi:10.2196/50225)
Supplement: Multimedia Appendix 1 [file jopm-v17-e50225-s001.pdf]

1) STUDY TITLE

Participant Engagement and Retention in Linkt (PEARL).

2) OBJECTIVES

The primary objective of this study is to evaluate effects of gamification and format of survey administration on participant retention, compliance, and engagement in a 6-month longitudinal study. The study will be structured as a siteless trial, in which brief (2-10 min) surveys will be administered remotely once per week in paper or electronic format.

To evaluate the primary objective, each participant will be assigned to one of four Arms, each consisting of two cohorts which will be recruited in sequence. Participants in each arm receive the surveys in a different format during the study (see table below). Participants in the cohorts will receive the same total compensation but on a different schedule. After enrolling approximately 50 participants each into the rapid payment cohorts (once every two weeks) of arms A, B, C, and D, or approximately 200 participants total, enrollment into the delayed payment cohorts (lump sum after 6 months) will begin.

Arm	Format of survey administration	Gamified format?	N (Approximate)	Payment Schedule
Arm A	Electronic: Smartphone app	Yes (opportunity to earn points, create custom avatar, track study progress on a map)	50	$5 per completed survey, paid every 2 weeks
			50	$5 per completed survey paid at 6 months
Arm B	Electronic: Datacubed Health website	No	50	$5 per completed survey, paid every 2 weeks
			50	$5 per completed survey paid at 6 months
Arm C	Non-electronic: Paper	No	50	$5 per completed survey, paid every 2 weeks
			50	$5 per completed survey paid at 6 months
Arm D	Electronic: Third-Party website	No	50	$5 per completed survey, paid every 2 weeks
			50	$5 per completed survey paid at 6 months

The secondary objective of the study is to assess the effect of gamification on participant satisfaction when performing cognitive tasks, as opposed to surveys. Participants who complete any of the initial 6-month cohorts will be recontacted and asked if they would like to continue into an optional, two-week follow-up period. Up to 400 participants will be recruited for this objective. Participants will then be re-randomized in the follow-up period. Participants will be

assigned (1:1) to one of two Arms, E and F. Arm E will examine participant satisfaction with non-gamified, web-based cognitive tasks. Arm F will examine participant satisfaction with gamified, app-based cognitive tasks. Satisfaction will be assessed using a standard, validated instrument, the Technology Acceptance Model (TAM) questionnaire delivered by web or app. Participants will receive $5 for each completed task or survey after the two-week follow-up period is complete, or up to $30 in compensation.

**Arm**	**Format of task administration**	**Gamified format?**	**N (Approximate)**
Arm E	Electronic: Smartphone app	Yes (opportunity to earn points, create custom avatar, track study progress on a map)	Up to 200
Arm F	Electronic: website	No (interactive, web-based tasks)	Up to 200

The specific aims of the study are:

Aim 1: Test whether electronic administration influences participant retention and compliance

<u>Hypothesis:</u> There will be a significant difference in retention and compliance between paper and electronic (web) administration of surveys. Retention will be measured by the number of days from the first assigned instrument to the date of the last fully completed instrument and compliance will be measured by the number of fully completed instruments. Electronic administration will improve retention and compliance, such that administration on paper will have lower retention and compliance compared to electronic administration on the non-gamified websites (Datacubed Health website and third-party website).

Aim 2: Test whether gamification influences participant retention and compliance

<u>Hypothesis:</u> There will be a significant difference in retention and compliance between gamified and non-gamified electronic administration of surveys. Gamification will improve retention and compliance, such that administration on the gamified app will have higher retention and compliance compared to administration on the non-gamified websites (Datacubed Health website and third-party website).

Aim 3: Test whether the format of survey administration influences the self-reported usability of the survey format

<u>Hypothesis:</u> There will be a main effect of the format of survey administration on self-reported usability of the survey format. Usability will be derived from a self-report usability survey that assesses the ease of use and pleasantness of the survey format; an overall measure of usability as well as different factors of usability will be derived from the survey responses.

Administration on paper will result in the lowest usability, and electronic administration on the gamified mobile app will result in the highest usability. Electronic administration on the non-gamified websites will result in intermediate usability, with use of Datacubed Health's website resulting in usability equivalent to or superior to a third-party website (i.e., paper < third-party website < Datacubed Health website < mobile app).

Aim 4: Test whether payment schedule influences retention and compliance differently by format of instrument administration.

Hypothesis: There will be a significant difference in retention and compliance between participants who receive biweekly payments and participants who receive one payment, adding up to the same amount, at study end. All participants will receive $5.00 for each completed survey. Participants who receive payment at study end will show reduced retention and compliance, and this will vary by format of survey administration (mobile vs. Datacubed web vs. third-party web vs. paper).

Aim 5: Test whether gamification of cognitive tasks influences participant experience and technology acceptance.

Hypothesis: Compared to participants who complete standard, non-gamified cognitive tasks (e.g., NIH Toolbox), participants who complete equivalent, gamified cognitive tasks within Datacubed Health's app will report a more positive experience on a usability/Technology Acceptance Model (TAM) questionnaire. Delivering cognitive tasks in a gamified format on mobile device will increase standard TAM outcomes (e.g., perceived ease of use, perceived usefulness, attitude towards using, behavioral intention to use).

Exploratory Aim 1: Test whether participant churn can be predicted from objective measures of participant engagement with the gamified smartphone app

Hypothesis: Participant churn (i.e. dropout) will be predicted by measures of participant engagement with the gamified smartphone app. This analysis will be restricted to Arm A participants assigned to the gamified smartphone app condition. Churn will be defined as missing more than 25% of assigned instruments within a study phase (the 1 month, 3 month, and 6 month milestones). Potential markers of engagement will included the number of clicks on the avatar, number of changes made to the avatar, number of gem store purchases, and the total amount of time spent on the app.

Exploratory Aim 2: Develop novel markers of participant behavior based on objective information from app use

Hypothesis: Geolocation data can be used to quantify and characterize the daily routines of participants, identifying markers consistent with self-reported activities and deviations from routine. Geolocation will be used to identify and classify spatiotemporal areas of interest and develop a data-driven model of daily routine. An activity tracker will be used to capture a self-report measure of daily activities. This analysis will be restricted to Arm A participants assigned to the gamified smartphone app condition.

3) BACKGROUND

The widespread availability and adoption of smartphones by people across all demographics and throughout the world has opened new and exciting frontiers of research that would have been unthinkable even ten years ago. The recent ubiquity of powerful computers with sophisticated graphics capabilities and built-in sensors that people carry with them all the time is beginning to transform research on health, behavior, and cognition. More and more research and clinical trials are going 'siteless,' opting to administer assessments remotely on mobile devices rather than in-person.

Missing data due to poor participant retention (i.e. dropout from the study and/or loss to follow-up) and compliance (i.e. missing or incomplete measurements) can jeopardize the scientific validity of studies and drive up the cost of conducting research and clinical trials. The National Academy of Sciences found patient dropout rates to be as high as 30% in clinical trials, prompting calls for better trial design and conduct to limit the impact of missing data[1]. Missing data can threaten the scientific validity of findings by introducing unintended bias and reducing statistical power, increasing the possibility that studies will fail to find effects where they exist (i.e. Type II errors)[2]. Siteless clinical trials may face additional challenges, since remote studies leave participants to their own devices and may eliminate direct contact with members of the research team.[3] Fortunately, technology and behavioral science offer an opportunity to address these challenges in novel ways by enabling more patient-centric practices to be implemented, which may ultimately accelerate research and drug development[4].

One promising non-financial, tech-enabled strategy for user engagement and retention that has emerged outside of clinical research is gamification: the application of game-like features such as points, avatars, and progress-trackers in a non-game context. Offering gamified platforms for research has received considerable attention as a potential alternative way to encourage participants to complete study activities that could augment or potentially even replace financial incentives. Gamification is hypothesized to be effective at engaging individuals by serving as an intrinsic motivational affordance, such that individuals are motivated by game elements because they satisfy intrinsic psychological needs for autonomy, relatedness, and competence.[5,6] While gamification of the user experience within smartphone apps for the collection of research data is both intuitively and theoretically appealing as a tool for incentivizing participation, no published study to our knowledge has directly tested whether gamification of a smartphone data collection system improves longitudinal participant retention and engagement. This study will address this knowledge gap.

An exploratory aim of the study is to develop novel objective measures of participant behavior based on data from phone sensors (specifically the geolocation, step-counter, and screen on/off times from the phone). Data-driven measures of behavior have great potential to enable better, more objective and sensitive assessments of health-related behaviors. For example, new objective measures of the amount individuals exercise (based on location and step counter data) or sleep (based on location and screen on/off data) would be useful for assessing changes in the health-related behavior of individuals following interventions as diverse as motivational interviewing, sleep medications, and dementia. Once established and validated,

sensor-based measures can be deployed directly onto individuals' phones, eliminating the need to collect or store raw geolocation data or other sensitive sensor-based information. (Please refer to attached whitepaper on location-based services for additional information.)

More sophisticated novel indices of behavior based on information such as phone location, step-counter output, and screen on/off data also have immense potential for health-related research and possible prediction of health outcomes. For instance, quantitative indices that characterize the regularity of people's daily routines, which can identify when an individual's activity begins to deviate abnormally from their own norm (e.g. when the individual begins spending much more or much less time at home than usual, or suddenly sleeps and wakes at irregular hours), could be useful both for measuring changes in behavior, and for predicting health outcomes. For example, among people with bipolar disorder, sudden changes in an individual's routine might presage an episode of mania or depression.[7,8] Detection of subtle early warning signs of an approaching behavioral episode or the onset of a disease using phone-based behavioral metrics would open the door to early interventions.

To test whether electronic and gamified tools delivered on smartphones can improve retention, compliance, and participant engagement, this study will directly compare retention and compliance between groups of participants assigned surveys administered in different formats: paper, two non-gamified web platforms, and a gamified mobile app. Additionally, we will evaluate the impact of gamification of standard, widely-used cognitive tasks (e.g., Stroop Task, Wisconsin Card Sort Task) on technology acceptance (e.g., perceived ease of use, perceived usefulness, attitude towards using, behavioral intention to use, etc).[9] The study will also examine an exploratory aspect of the study, prediction of participant dropout (i.e. churn), at different phases of the study using objective markers of engagement with the gamified mobile app. We plan to further leverage the unique capabilities of mobile devices in the gamified mobile app group to gather phone-based behavioral data (i.e. geolocation, step-counter, screen on/off times, and battery life) and develop novel behavioral metrics (e.g. quantitative measures of daily routine regularity), which we will validate relative to the self-reported information from surveys. Smartphone-based data collection opens unique opportunities to address retention and compliance, identified as major problems for clinical trials[10], and enables collection of denser longitudinal data and derivation of novel measures from sensor data.

References

1. National Research Council (US) Panel on Handling Missing Data in Clinical Trials. The Prevention and Treatment of Missing Data in Clinical Trials. Washington (DC): National Academies Press (US); 2014.
2. Fewtrell, M. S. *et al.* How much loss to follow-up is acceptable in long-term randomised trials and prospective studies? *Arch. Dis. Child.* **93**, 458–461 (2008).
3. National Research Council, Division of Behavioral and Social Sciences and Education, Committee on National Statistics & Panel on Handling Missing Data in Clinical Trials. *The Prevention and Treatment of Missing Data in Clinical Trials*. (National Academies Press, 2011).

4. Sharma NS. Patient centric approach for clinical trials: Current trend and new opportunities. Perspect Clin Res. 2015;6: 134–138. 4.
5. Zhang, P. Motivational Affordances: Fundamental Reasons for ICT Design and Use. *Commun. ACM*
6. Deci, E. L. & Ryan, R. M. *Handbook of Self-determination Research*. (University Rochester Press, 2004).
7. Beiwinkel, T. *et al.* Using Smartphones to Monitor Bipolar Disorder Symptoms: A Pilot Study. *JMIR Ment Health* **3**, e2 (2016).
8. Grünerbl, A. *et al.* Smartphone-based recognition of states and state changes in bipolar disorder patients. *IEEE J Biomed Health Inform* **19**, 140–148 (2015).
9. Wu, J., Wang, S. What drives mobile commerce? An empirical evaluation of the revised technology acceptance model. *Information & Management*. **42**, 719-729 (2005).
10. Tudur Smith C, Hickey H, Clarke M, Blazeby J, Williamson P. The trials methodological research agenda: results from a priority setting exercise. Trials. 2014;15: 32.

4) INCLUSION AND EXCLUSION CRITERIA

Participants must be 18-89 years of age, reside in the United States, have access to Google Chrome or Safari internet browser (on a computer, tablet, or phone) and email, and be able and willing to complete and return one to two surveys per week. Participants must also own an Android or iOS smartphone (manufactured in 2014 or later) with an operating system capable of supporting the study app (Android 6.0 or greater; iOS 10.0 or greater) and with an active data plan (1GB/month or more). Potential participants will be asked to indicate that they understand English well enough to comprehend the informed consent documents and study instructions. English language abilities will be confirmed using comprehension questions during the consent process (see Section 29). No individuals unable to demonstrate they are capable of giving informed consent in English by correctly answering these questions will be enrolled. During consent, we will ask participants if they have any prior experience with the study smartphone app. Participants who indicate prior experience with the study smartphone app will be excluded from the study and will be considered ineligible.

We will not collect information about pregnancy or exclude subjects based on pregnancy status because the study will only involve use of a smartphone app and no other interventions or procedures. We will not recruit prisoners. We will not recruit minors under the age of 18.

5) STUDY-WIDE NUMBER OF SUBJECTS

We plan to enroll up to 400 individuals in the study (see Section 9).

6) STUDY-WIDE RECRUITMENT METHODS

Potential participants are expected to be recruited over the course of one year, starting at the end of Q3 2020 or start of Q4 2020. An advertisement in the Metro newspaper will be placed to recruit potential participants; both a print ad (in the physical print Metro newspaper in New York City) and an electronic ad (on Metro's website) will be placed. Electronic ads will be placed in

the New York City page of the Craigslist website, specifically in the ‘Volunteers’ section. Electronic ads will be posted on Facebook and Instagram, and links will be posted on Facebook and Reddit groups that include primarily adult participants located in the United States. The study will also be posted to online participant recruitment platforms including, but not limited to, User Interviews and Autocruitment. The print Metro newspaper is free and accessible from distribution racks placed inside 100 New York City subway stations; the websites and social media platforms are free and accessible to anyone with an internet connection.

The source of participants will be people who self-report living in the United States who meet one of the following criteria;

- Read the physical print Metro newspaper or the Metro website
- Use Craigslist, Instagram, Facebook, or Reddit.
- Are part of third-party websites (e.g., User Interviews, Autocruitment) which function to connect participants with user research studies.

A copy of the ad (print and online) is included as an attachment in this application. The ads include the following information:

- Description of Datacubed Health
- Description of the study design + objectives
- Eligibility requirements for study participation
- Study participation requirements: completion of approximately one survey per week
- Compensation for study participation: $5 for each survey or task completed
- Contact information for Datacubed Health (link to website)

Potential participants who are interested in the study will visit the website listed on the study ad. This website will be hosted on Datacubed Health’s secure servers and will contain additional information about the study. The complete study website text is included as an attachment. Those who review the information on the website and wish to participate will be directed to an online screening questionnaire. The study team will then invite those potential participants whose screening questionnaires indicate that they meet study inclusion criteria to review and sign the study consent form online through Datacubed Health’s web-based platform (see Section 29 for further details on consent process).

Datacubed Health will inform potential participants whose screening questionnaires indicate that they do not meet study inclusion criteria that they have not been selected to participate in the study.

7) STUDY TIMELINES

Each participant will be asked to complete surveys for six months after enrollment. Over the course of that six-month period, they will receive roughly one survey each week either by mail, within the smartphone app, through the Datacubed Health website, or through a third-party

website. Each survey will last no longer than 10 minutes, but most will be shorter (~3 minutes). A subset of participants who complete the initial six-month period will be given the option to continue into a two-week follow-up period, during which they will complete up to 6 more activities (cognitive tasks and usability/TAM surveys).

We plan to enroll all participants in the study within one year. We plan to complete primary analyses of the study data within two years.

8) STUDY ENDPOINTS

The primary outcomes of interest are measures of *retention, compliance,* and *usability*.

- *Retention* will be defined as the amount of time participants remain active in the study. Specifically, the primary retention measure will be the number of days between the participant's first assigned study instrument and the date of the last assigned study instrument that they complete.
- C*ompliance* will be defined as the number of instruments that the participant fully completes (i.e. responses for all required items) over the course of the study.
- *Usability* will be derived from a survey-based measure that assesses the perceived ease of use and pleasantness of the survey format (see separate attachment). The mean score across items of the survey measure will be used as the primary usability outcome measure of the overall usability of the survey format.

The exploratory outcome measures of interest are measures of *churn, engagement, activities,* and *routine*:

- *Churn* will be defined as missing more than 25% of assigned instruments within a study phase. Study phases will be defined at the 1 month, 3 month, and 6 month milestones.
- *Engagement* will be assessed based on participant behaviors while using the gamified mobile app (Arm A), and will include the number of clicks on the avatar, number of changes made to the avatar, number of gem store purchases, and the total amount of time spent on the app.
- *Activities* will be defined by classification of clusters of spatiotemporal geolocation data (Arm A).
- *Routine* will be defined by a directed graph model of the *activities*; characteristics will be explored using graph theoretic measures (Arm A).

9) STUDY DESIGN AND PROCEDURES

Arms A-D

This study will employ at initial recruitment a between-subjects design with four, independent arms, divided into two sequential cohorts.

Each participant will first complete the informed consent process (see Section 29). Upon receipt of the signed consent (signature captured digitally), Datacubed Health will assign the participant

to one of 4 groups; group assignment will determine the medium through which the surveys will be administered -- gamified mobile app (Arm A), web browser (Arm B & Arm D), or paper (Arm C). Approximately 100 participants are expected to be enrolled into each group.

Group assignment will be done sequentially by date and time of consent signature – i.e., the first participant who signs consent will be assigned to Arm A; the second participant, to Arm B; the third participant, to Arm C; the fourth participant, to Arm D; the fifth participant, to Arm A; the sixth participant, to Arm B; etc. The first cohort of participants recruited into each arm will receive compensation every two weeks based on how many instruments they complete ($5/instrument). The second cohort of participants recruited into each arm will receive a single payment at the conclusion of the 6-month arm. Compensation will be the same, $5/instrument, but delivered as a single lump sum payment after completion. The maximum, total compensation possible in the initial 6-month study period will be $130.

Each participant will be informed immediately of their group assignment and compensation scheme. Participants who are assigned to the mobile app group will receive additional instructions on how to download and log into the app. Participants who are assigned to the Datacubed Health website group will receive an email with instructions and a link to log into the Datacubed Health web portal. Participants who are assigned to the third-party website (Qualtrics) will be informed that they should expect to receive an email with a custom link to complete surveys each week. The third-party website will not be provided with any participant information, including email addresses. Subjects will only be identified to the third-party website with unique, randomly generated alphanumeric IDs. A script hosted by Datacubed Health will link these IDs to participant email addresses, internally. A master list of alphanumeric subject IDs and participant email addresses will be visible only to study administrative staff at Datacubed Health. Participants who are assigned to the paper group will be informed that they should expect to start receiving their surveys by mail each week.

Participants in all groups will be assigned the same set of study instruments, in the same order. See separate attachments for a listing and full text of all planned survey instruments. Each instrument will take no more than 10 minutes to complete (including embedded instructions), but most will be substantially shorter (2-3 minutes).

Participants in the gamified mobile app condition must log into the mobile app and will see a rich user interface - this includes a customizable avatar, progress-tracking maps that are gradually revealed as study activities are completed, points earned by completing study activities that can be redeemed for virtual avatar accessories (e.g. a hat or sunglasses), and other appealingly designed features (e.g. animated confetti displayed when study activities are completed). Participants in the non-gamified web conditions (Datacubed Health and third-party) must log into the respective web platform and will simply see a scrollable list of available study activities. Participants in the paper condition will be mailed paper surveys, which they must return to the study team through mail using pre-addressed and stamped envelopes. Screenshots illustrating examples of each study condition are provided as a separate attachment.

After 6 months, participants who indicate that they wish to continue with the study will enter an optional two-week, follow-up period. Participants will be randomized to complete activities (cognitive tasks and usability/TAM questionnaires) in the same smartphone app used for the initial 6-month, study period, or using a secure email link. Participants will complete validated, commonly used cognitive tasks assessing working memory and decision-making (e.g., Stroop Task, Wisconsin Card Sort Task, N-Back task, etc.). After completing each cognitive task, participants will complete a brief usability/TAM questionnaire asking about their experience completing the relevant task. Participants in the app group will complete the usability/TAM questionnaire in the app, while participants in the web group will receive a separate secure email link to complete the same questionnaire.

Participants will receive $5 for each week of the study if the study surveys for that week are completed. For the first cohort of 50 participants in each arm, reimbursements will be processed on a regular schedule (every other week) using PayPal, a third-party electronic payment system. For the second cohort of 50 participants in each arm, reimbursements will be processed once, after 6 months of participants. Participants who complete the optional two-week follow-up will receive $5 via PayPal for each activity completed, after two-weeks. Participants will need to set up a PayPal account. This will require providing certain PII such as name, email address, and checking account information to PayPal and agreeing to the PayPal's privacy and security policies. Datacubed Health will require minimal PII (name and email address) to send payment to the participants through PayPal. These PII will be handled as described immediately below.

Certain personal identifiable information (PII), such as name, email address, and phone number, will be collected from all participants; mailing addresses will also be collected for participants in the paper group. These data will be used for administrative purposes only (e.g. contacting participants and sending surveys). For security, these data will be encrypted and stored in separate files, which can only be referenced through the anonymous subject ID that is assigned to each participant for the study. These data will be destroyed at the end of the study.

Certain demographic data, such as age (month and year of birth only; all subjects are under 90), gender, sex, ethnicity, education level, employment status, annual income level, and type of smartphone, will be collected from all participants. These data will be analyzed to see if they are associated with any differences in retention, compliance, and engagement. For security, these data will be encrypted and stored in separate files, which can only be referenced through the anonymous subject ID that is assigned to each participant for the study.

Survey data, including survey responses, number of surveys completed, and dates of survey completed will be collected for all participants. These data will be used to evaluate the study endpoints of participant retention, compliance, and usability.

In addition to the aforementioned data types, for the group assigned to the gamified mobile app, several types of data provided by the phone operating system will be collected:

- Passive data: These types of data will be collected even when the participant is not actively using the app. Specifically, the app will record information about the participant's

phone battery percentage, geolocation, number of steps taken, apps installed on the phone, and phone screen on/off times. Participants will be fully informed during the consent process that the app will collect such data. They will be able to disable collection of location and step count data at any time by restricting the app permissions via the phone operating system; Android phone owners will also be able to restrict permissions for data collection on screen time.

    - Geolocation is the collection of precise geographical location (latitude and longitude) of a device through a mixture of GPS and WiFi signals. This technology is used by many other apps that provide services based on a user's location, such as navigation, rideshare, banking, dating, and fitness apps. When location permissions are allowed, location data is sent to the app every few minutes. In this study, geolocation data will only be used to develop a data-driven model of participants' daily routines. This model will then be compared with self-reported measures of daily activity to see if geolocation data can serve as a measure of activity and routine. Datacubed Health will not share geolocation data with any other external parties and will destroy this data at the end of the study.
    - Once the data collection phase of the study is complete, participants will be automatically logged out from the app and all forms of passive data collection will cease. Additionally, most phone operating systems will not share geolocation data when the phone is in battery save mode and after the app has not been used by the user for a set period of time (length determined by the operating system).
- App behavior data: When a participant is using the app, data on the participant's behaviors within the app (e.g. which buttons are clicked, how frequently buttons are clicked, how long participants spend on certain screens, etc) will be collected.
- These passive data and app behavior data will be used to develop novel objective measures of participant behavior.

Arms E and F

At the end of the initial 6-month participation period, all participants will be asked if they would like to continue into an optional two-week, follow-up period. At the end of this period, participants will be asked if they would like to be contacted about other research opportunities. Participants who complete PEARL study measures, and agree to be contacted, may be contacted in the future, via email, as part of recruitment efforts for other projects.

10) DATA AND SPECIMEN BANKING

No specimens will be collected in this study.

When the study ends and we close out the study with the IRB, we will purge all personally identifiable data except location data (see section 26). While we may use the resulting deidentified data for internal product development purposes, we do not plan to release the data; access to the deidentified data will remain limited to members of Datacubed Health who have received CITI training in the handling of human subjects data. We will never release personally identifiable information (including raw location data) outside the organization.

11) DATA MANAGEMENT

Participants in the mobile app group will use unique, coded credentials to log in to the study app. For each participant, we will generate a random ID code (a random alphanumeric string) and password. During the initial login, participants will have the option to use their phone's built-in biometric identification features (fingerprint or face ID) to log into the app in the future. All research data collected by the app will be encrypted on the device, and the participant will not be able to access or read the encrypted data using their credentials. We will not have access to the biometric signatures used by the smartphone operating systems.

Periodically, when a network connection is available, the app will upload data to an encrypted server using an encrypted (and HIPAA compliant) transmission protocol. As part of the upload process, the data will be validated as uncorrupted, then deleted from the phone. Research data stored on the encrypted server will be coded using the participant's random alphanumeric ID code, and it will not be directly linked to personally identifiable information (PII) during data storage or analysis, except insofar as geolocation data may itself contain identifying information. Access to the encrypted and coded data stored on the server will be password-protected, and it will only be accessible to authorized, human subjects trained, personnel within Datacubed Health. Data will be maintained on the server for the duration of the study. The data will only be analyzed in a coded or de-identified form. De-identification in this case refers to the removal of ID codes.

Participants in the web groups will use unique, coded credentials to log in to the web platforms. For each participant, we will generate a random ID code (a random alphanumeric string) and password. All research data collected by the web platforms will be encrypted, and the participant will not be able to access or read the encrypted data using their credentials.

Each participant in the paper group will be assigned a random ID code (a random alphanumeric string) which will be used to identify his/her data when the paper surveys are transcribed into electronic format for data analysis.

Ongoing quality assurance checks will be performed throughout the study, to identify any invalid or missing data and to address the possible reasons for such problems.

<u>Data analysis plan:</u>

Once data collection is complete for all participants, we plan to perform independent t-tests on the outcome measures of retention and compliance and a 1-way ANOVA on the outcome measure(s) of engagement. See Section 8 for a description of how each measure will be calculated.

Aim 1: Test whether electronic administration influences participant retention and compliance

To test whether electronic administration influences participant retention and compliance, we will perform a one-way analysis of variance (ANOVA) with Dunnett's test for multiple comparisons to assess retention and compliance between the groups receiving paper-based administration of surveys and the two groups receiving electronic administration on the non-gamified websites. Non-parametric alternatives (e.g., Kruskal-Wallis test) may be used in place of ANOVA if the data significantly violate the assumptions for ANOVA. This approach will eliminate the potential confounding effect of gamification on the outcome measures by excluding the group receiving electronic administration of the gamified mobile app.

Aim 2: Test whether gamification influences participant retention and compliance

To test whether gamification influences participant retention and compliance, we will perform one-way ANOVA with Dunnett's test for multiple comparisons to assess retention and compliance between the groups receiving electronic administration on the non-gamified websites and the group receiving electronic administration on the gamified mobile app. Non-parametric alternatives (e.g., Kruskal-Wallis test) may be used in place of ANOVA if the data significantly violate the assumptions for ANOVA. This approach will eliminate the potential confounding effect of electronic administration of surveys on the outcome measures by excluding the group receiving paper-based administration of surveys.

Aim 3: Test whether the format of survey administration influences the self-reported usability of the survey format

To test whether the format of survey administration influences self-reported usability of the survey format (Aim 3), we will perform one-way ANOVA to compare overall usability between all four groups. The independent variable in the ANOVA model will be *survey format* (levels: paper non-gamified, web non-gamified (Datacubed Health), web non-gamified (third-party website), and mobile app gamified). We will perform follow-up pairwise comparisons using a Tukey's Honest Significant Difference (HSD) post-hoc test to correct for multiple comparisons and identify the source of any significant *F*-scores from the ANOVAs (i.e. to test whether a significant main effect of app version is driven by differences between the non-gamified paper version vs. non-gamified web versions, non-gamified paper version vs the gamified mobile app version, the Datacubed Health website vs. the third-party website, or the non-gamified web versions vs. the gamified mobile app version). Factor analysis will also be applied to the responses from the usability survey to assess different factors that contribute to the overall usability outcome measure; if stable factors are identified they will be assessed using the approach outlined above.

Aim 4: Test whether payment schedule influences retention and compliance.

To test whether payment schedule influences retention and compliance (Aim 4), we will perform a two-way ANOVA to compare overall retention and compliance between groups (Arms A vs. B vs. C vs. D) and payment schedule (payment biweekly vs. payment at study end). A significant interaction effect is expected, such that differences in retention and compliance between arms will be more pronounced among participants receiving one payment at the end of the study.

Aim 5: Test whether gamification of cognitive task administration influences participant experience and technology acceptance.

To test whether the format of cognitive task administration influences participant experience and technology acceptance, we will compare responses on the usability/TAM surveys between groups (app vs. web task administration) using between subjects t-tests.

Exploratory Aim 1: Test whether participant churn can be predicted from objective measures of participant engagement with the gamified smartphone app

An exploratory aim of the study is to identify objective measures of participant behavior on the gamified mobile app that predict retention, compliance, and engagement. Based on the behaviors of participants in the gamified mobile app group, we will use logistic regression and a random forest classifier to perform a survival analysis to predict participant "churn" (i.e. whether or not participants are retained at a given stage of the study). Churn will be predicted for the 3-month and 6-month milestones, based on participant behavior in the previous milestone (respectively at the 1-month and 3-month milestone). Features will include potential markers of engagement including the number of clicks on the avatar, number of changes made to the avatar, number of gem store purchases, and the total amount of time spent on the app. The importance of each feature will be explored to inform which features of the app most engage participants. The logistic regression and random forest classifier models will be compared based on their accuracy, precision, and the F1-score. A confusion matrix will be used to visualize the performance of the supervised learning algorithms; each row of the matrix represents the instances of the predicted class while each column represents the instances of the actual class. A range of thresholds (i.e. the probability threshold used to determine churn) will be explored to tune the accuracy and precision of the models.

Exploratory Aim 2: Develop novel markers of participant behavior and engagement based on objective information from app use

Another exploratory aim of the study is to develop novel objective measures of participant behavior based on data from phone sensors (specifically phone location, step-counter, and screen on/off times). As noted in Section 3, such measures hold enormous potential to revolutionize the measurement of human behavior via data-driven assessment, as well as the prediction of health-related outcomes. Therefore, we plan to analyze phone sensor data collected in this study to further develop data-driven behavioral measures we have begun to develop using internal pilot data, and to validate those measures relative to self-reported information about individuals' behavior (based on the activity tracker).

As an example, we plan to continue development of an algorithm to quantify individuals' daily routines using a graph-theoretic approach based on mobility data (location & step-counter) and screen on/off data. First, these data will be preprocessed (normalized, resampled, and interpolated as necessary). Next, unsupervised clustering algorithms (e.g. DBSCAN) will be used to identify spatiotemporal patterns in the data (i.e., locations that the individual tends to frequent at regular times). Spatiotemporal areas of interest identified via the clustering analysis,

and periods of interstitial data between them (e.g. periods of travel from one location to another) will then be classified into different types of events using standard machine-learning techniques (e.g. random forest classification, neural networks). Then, we plan to transform the resulting information into an abstracted directed graph model of the individual's typical daily routine. Once a model of an individual's typical routine is established in this data-driven way, it can be used to quantify the amount and type of deviations from the routine captured in subsequent data using standard graph-theoretic metrics. In this study, we will further refine and develop these and other metrics for the data-driven characterization of behavior, then validate the accuracy of the resulting metrics using ground-truth data from the self-report activity tracker.

12) PROVISIONS TO MONITOR THE DATA TO ENSURE THE SAFETY OF SUBJECTS

This study involves no additional risk to the subjects beyond what is encountered in daily life. There are no plans to monitor the safety of the subjects. If we receive information suggesting that a subject is at risk, they will be encouraged to call 911 or contact an emergency medical provider.

13) WITHDRAWAL OF SUBJECTS

Subjects are free to withdraw at any time. They do so by contacting the study team by email. The study team will then delete their credentials from the Datacubed Health system after which the participant would no longer be able to access the app or websites beyond the initial login screen, and both the app and website would be unable to transmit any further data to the server. Subjects who were receiving their paper surveys by mail would not receive any subsequent surveys by mail, and the only further study-related contact required with the subjects would be to process any pending payments to date.

Subjects may also withdraw from the study by ceasing to return surveys. This will be identified as non-compliance rather than as a withdrawal.

When a subject withdraws from the study all data collected prior to withdrawal is retained. Subjects are informed of this at the time of consent.

14) RISKS TO SUBJECTS

We do not anticipate any direct physical risks to the participant.

Risks to the subjects are minimal and do not exceed those encountered in daily life. Subjects may experience eye strain normally associated with reading and writing. Subjects will be informed that if they experience any discomfort of this kind they should cease the activity and rest before returning to the study-related task.

Participants may experience some psychological discomfort when answering survey questions that ask about thoughts, mood, or behaviors. Subjects are free not to answer any questions that they find uncomfortable.

The primary risk associated with this study is that an unauthorized party could gain access to study data and use it to identify participants. However, we have taken many precautions to ensure that this will not happen. The software systems we will use are built to protect the security of sensitive study data (see Sections 11 and 25). We will use safeguards such as strong encryption for all data on participants' phones, in transit to our servers, and on our servers. All access to study data will be password-protected and limited to authorized members of the study team. Other personnel involved in the development and maintenance of the software (e.g. the server administrator) will only have access to non-sensitive metadata (e.g. information about the date and time of failed data transmissions). Although we will do everything in our power to ensure the continued privacy and security of data from the study, unauthorized or unintentional disclosure of such data remains a minimal risk.

Geolocation data can also carry privacy risks for participants when the use, sharing, and handling of location data are not fully disclosed to participants. During the consent process, participants will be informed that the only use of geolocation data in this study is for comparison with self-reported survey data (i.e., to determine whether geolocation could serve as a novel measure of activity and routine); that the data will not be shared with any external parties; and that the data will be destroyed at the end of the study. Geolocation data will be handled in the same manner as PII.

15) POTENTIAL BENEFITS TO SUBJECTS

There is no benefit to the subjects from participation.

16) VULNERABLE POPULATIONS

We will not recruit minors or prisoners. We will not exclude pregnant women, so pregnant women could be included in our cohort. We will not solicit or record any information about whether one of our subjects is pregnant.

17) MULTI-SITE RESEARCH

This is a siteless study. Administration of the study and research team activities will occur at a single site: Datacubed Health.

18) COMMUNITY-BASED PARTICIPATORY RESEARCH

Not applicable.

19) SHARING OF RESULTS WITH SUBJECTS

No results will be shared with subjects.

20) SETTING

This is a siteless study; participants will be recruited via paper and internet advertisement (see section 6) and they will never visit a physical location to perform any study activities. The research team will be based at the offices of Datacubed Health.

21) RESOURCES AVAILABLE

Datacubed Health will provide the resources necessary to conduct this study, including the necessary infrastructure and financial support. (Subjects will use their own smartphones, pencils/pens, and computers, as applicable.) The organization will provide access to and support for the app. Datacubed Health will provide compensation to those participants as they are assigned to receive it. Participants will earn $5 for each week of successful survey completion; participants can thus earn anywhere between $0 (if no surveys completed at all) to $130 (if surveys are successfully completed over 6 months, or 26 weeks). Payments will be processed and distributed to participants every other week, for the first cohort of 50 participants enrolled in Arms A, B, C, and D (approximately 200 participants). Payments will be processed after 6 months for the second cohort of 50 participants enrolled in each arm (approximately 200 participants). Participants who choose to enter the optional two-week follow-up period will receive one PayPal transfer consisting of $5 for each survey or task completed in that time, after two weeks.

The study team, who will be based at the offices of Datacubed Health, is composed of multiple well-qualified full-time researchers who will devote the time necessary to conduct and complete the study. Members of the team include:

- Marie Onakomaiya (PI), a PhD level behavioral neuroscientist with a master of public health and over 10 years of research and evaluation experience, including in clinical and population health, as well as survey design, implementation, and analysis.
- Elias Boroda (Research Scientist), a PhD level behavioral neuroscientist with XX years of research and clinical trial experience
- Silas Ryan (Associate Research Scientist), a BS level scientist with graduate-level coursework in biostatistics and epidemiology and 2 years of experience developing and testing methods for mobile data collection
- Michelle Cinguina (Assistant Research Scientist), a BS level scientist with three years of experience coordinating clinical research trials, including in digital health.

All members of the research team have been involved in the design of the study and will have access to the IRB-approved protocol. The PI will take primary responsibility for the conduct of the study and will delegate specific duties to other members of the study team as necessary.

Since participants will be recruited from the New York City area, the inclusion criteria for the study are very broad, and the enrollment process is mostly automated, we believe it is entirely feasible to enroll the intended 400 participants within one year.

22) PRIOR APPROVALS

No prior approvals are required.

23) RECRUITMENT METHODS

Please refer to Section 6 for recruitment methods.

24) LOCAL NUMBER OF SUBJECTS

This is a siteless trial. There will be no distinction between local and non-local subjects.

25) CONFIDENTIALITY

Participation in the study will be confidential. We will never identify individuals as participants in the study, and we will use a multi-pronged approach to ensure the privacy and security of participant data so that no other party can ever identify individuals as participants in the study (see Sections 11 and 26).

26) PROVISIONS TO PROTECT THE PRIVACY INTERESTS OF SUBJECTS

Research data will be coded, access-protected, and encrypted both at rest and in transit to ensure data privacy and security (see Section 11 - Data Management for details).

Some personally identifiable information (PII) such as name, email address and phone number must be collected from participants during the screening process for administrative purposes: this information will enable us to contact the participant when necessary and to enable reimbursement payments. This PII will not be directly linked to research data about the participants on the server, and the coded data will never be linked to these PII during data analysis. A table linking PII to participants' coded IDs will be kept in a separate encrypted and password-protected database that will only be accessible to study personnel. At the conclusion of the study, when the closure of the study is approved by the IRB, we will permanently delete the table containing the administrative PII.

Throughout the entire study, and after, we will also take additional precautions to ensure that geolocation data collected by the app cannot be used to identify participants. We will never publish nor otherwise disseminate raw geolocation data. We will only publish or otherwise disseminate geolocation data that is not identifiable (e.g. short snippets of data that are not linked to a particular date and time, or summary data that are aggregated over time and/or participants).

27) COMPENSATION FOR RESEARCH-RELATED INJURY

Not applicable; this is a minimal risk study.

28) ECONOMIC BURDEN TO SUBJECTS

For participants in the mobile app group, the study app and all assessments within it will be provided at no charge. However, each participant will need to install the study app on his or her own Android or iOS smartphone with a data plan. Nearly all phones produced within the past

few years will support the app. However, if a prospective participant does not own such a phone, they will not be eligible for participation.

The study app can transmit data over Wi-Fi and cellular connections. Depending on the participant's data plan, it is possible that a participant could incur charges for data sent via the cellular network. However, we expect that the amount of data sent by the study app will be minimal relative to many commonly used apps (e.g. games, social networking, email apps). Therefore, we anticipate that for most participants this cost will be minimal or nonexistent, compared to the data transmission costs they incur under normal circumstances. Participants will be able to further mitigate any potential costs by connecting to Wi-Fi when available. At the time of consent, this potential cost will be clearly explained to the subjects.

For participants in the web groups, the web platforms and all study assessments within them will be provided to participants at no charge. However, participants will need to procure their own internet access and hardware (e.g. smartphone, computer, or tablet) for using the internet.

For participants in the paper group, paper surveys will be mailed to participants at no charge. Each survey that Datacubed Health mails will also include a return envelope with prepaid postage.

29) CONSENT PROCESS

Written consent will be obtained for this study. Potential participants who are interested in the study and who have met study eligibility criteria will be asked to provide consent electronically via Datacubed Health's secure, HIPAA-compliant web-based platform.

For each participant, the following steps for the consent process will be followed:

- One of the study investigators (PI or Sub-Is) will add the participant to Datacubed Health's database by entering the name and email address of the participant.
- The Datacubed Health database will generate and send an email to the participant containing a link to the secure web platform and unique temporary log-in credentials.
- Using the link and temporary log-in credentials received by email, the participant will log into the web platform. The participant will update his/her password and will then be prompted to review the study consent form.
- The participant will be able to read the consent form in its entirety. To facilitate understanding and comprehension, a short summary of certain sections will be provided to re-iterate important points in succinct, clear language.
- The consent form will contain contact information for Datacubed Health (email and phone number). If the participant has any questions or concerns as a result of reviewing the consent form, he/she can contact Datacubed Health to further discuss before signing the consent form. One of the study investigators (PI or Sub-Is) will contact the participant within 1-2 business days to discuss the participant's questions or concerns directly.
- After the participant has read the consent form, the participant will need to answer several comprehension questions (see separate attachment) to ensure that he/she has

understood the form and is able to give informed consent. If the participant answers all comprehension questions correctly, he/she will be given the opportunity to sign the consent form. If a participant does not answer the comprehension questions correctly, he/she will be redirected back to review the consent form content, then will have the opportunity to complete the comprehension questions again. This process will be repeated such that each participant will have a total of 3 chances to correctly complete the comprehension questions. If the participant is unable to correctly complete the comprehension questions after 3 attempts, the participant will be removed from consideration for the study.

- The participant will sign the consent form in the web platform. The signature field in the web platform allows for the participant to physically represent his/her actual signature using a finger or stylus (if using a tablet or laptop with trackpad or smartphone) or a mouse (if using a computer). Physical signatures will thus be required and will be digitally captured.
    - Please refer to Section 30 for further details regarding documentation of written consent using an electronically captured signature.
- Once the participant electronically signs the consent, a dated, timestamped PDF copy of the consent form with his/her printed name and the image of his/her signature in the signature field will be generated by the server. A PDF copy of the consent form will automatically be emailed to the participant, and another copy will be stored on the secure server.

Participants who choose to enter the optional, two-week follow-up period will complete an addendum to the informed consent form, using the same platform for initial consent and following the same procedures.

If any prospective participant declines to provide consent, they will not be enrolled in the study and they will be unable to access any features of the web platform apart from the eConsent module.

Only participants who are able to comprehend written English will be enrolled in this study because all consent and study materials will be provided to participants in English.

There will be no assent process in this study, as only adult participants between the ages of 18-89 years will be enrolled. During eligibility review, potential participants will be asked to provide their month and year of birth to confirm their age.

As part of the eligibility review, Datacubed Health will ask individuals whether they are able and willing to meet the study requirements of completing and submitting one to two surveys per week. Datacubed Health will also evaluate whether each participant demonstrates the ability to respond to the study ad and correspond with Datacubed Health to consent to the study.

The study will recruit adult participants who personally give consent and sign the consent themselves. The study will not necessitate permission from legally authorized representatives.

As this is neither a HUD nor an investigational drug study, there are no potential risks or benefits associated with HUD or drug to be described in the consent.

30) PROCESS TO DOCUMENT CONSENT IN WRITING

HHS OHRP and FDA regulations both permit the use of electronically captured signatures when written informed consent is required.

In the proposed study, subjects will review and sign the study consent form through Datacubed Health's secure web-based platform. This platform adheres to 21 CFR Part 11 standards for electronic records and electronic capture of signatures, and is considered to be an equivalent to a handwritten signature executed on paper.

The attachment titled "FDA CFR Title 21 Part 11 Compliance Documentation Log" is included in this proposal as documentation to support the use of Datacubed Health's web-based platform to document consent in this study; full details are provided in this file, but some key features include:

- Closed system controls to ensure accuracy, reliability, and validity of electronic records and prevent unauthorized access or modifications to electronic records
- Distinct identification components (user ID and participant authentication token) to verify that each electronic signature belongs to its genuine owner
- Capture of the date and time of signature and the printed name of the signer are captured in the audit trail

31) DRUGS OR DEVICES

This study does not involve any drugs or devices.
